# Variations in Estimated Glomerular Filtration Rate Across Countries in Patients With Metabolic Dysfunction‐Associated Steatotic Liver Disease and Their Association With Liver Fibrosis: A Multicenter Study

**DOI:** 10.1002/mco2.70503

**Published:** 2025-11-24

**Authors:** Jing Zhao, Ferenc E. Mózes, Xin‐Yu Xu, Dong Ji, Huiqing Liang, Xiaoling Chi, Jinjun Chen, Takeshi Okanoue, Toshihide Shima, Yongfen Zhu, Christian Labenz, Bihui Zhong, Masato Yoneda, Atsushi Nakajima, Junping Shi, Jing Zhang, Sanjiv Mahadeva, Wah‐Kheong Chan, Fangping He, Chun‐Yan Ye, Su Lin, Adèle Delamarre, Victor de Lédinghen, Monica Lupsor‐Platon, Zhonghua Lu, Hong Tang, Jidong Jia, Peter J. Eddowes, Liang Xu, Yiling Li, Yuemin Nan, Hong Deng, Junqi Niu, Xuebing Yan, Qing Ye, Qinglei Zeng, Yongjian Zhou, Jeremy F. L. Cobbold, Chenghai Liu, Jie Li, Lei Li, Jing Wang, Fanpu Ji, Jin Chai, Yongning Xin, Giovanni Targher, Christopher D. Byrne, Yuchen Fan, Jia‐Hui Zhang, Geraldine Ooi, Jacob George, Michael Pavlides, Dan‐Qin Sun, Ming‐Hua Zheng

**Affiliations:** ^1^ Urologic Nephrology Center Jiangnan University Medical Center Wuxi China; ^2^ Affiliated Wuxi Clinical College of Nantong University Wuxi China; ^3^ Department of Urology Wuxi No.2 People's Hospital Wuxi China; ^4^ Oxford Centre for Clinical Magnetic Resonance Research Division of Cardiovascular Medicine Radcliffe Department of Medicine University of Oxford Oxford UK; ^5^ Senior Department of Hepatology The Fifth Medical Center of Chinese PLA General Hospital Beijing China; ^6^ Hepatology Unit Xiamen Hospital of Traditional Chinese Medicine Xiamen Fujian China; ^7^ Department of Hepatology The Second Affiliated Hospital of Guangzhou University of Chinese Medicine Guangdong Provincial Hospital of Chinese Medicine Guangzhou China; ^8^ Hepatology Unit Department of Infectious Diseases Nanfang Hospital Southern Medical University Guangzhou China; ^9^ Department of Gastroenterology and Hepatology Saiseikai Suita Hospital Suita Japan; ^10^ Department of Hepatology and Infection Sir Run Run Shaw Hospital Affiliated With School of Medicine Zhejiang University Hangzhou China; ^11^ Department of Internal Medicine I University Medical Centre of the Johannes Gutenberg‐University Mainz Mainz Rhineland‐Palatinate Germany; ^12^ Department of Gastroenterology the First Affiliated Hospital Sun Yat‐sen University Guangzhou China; ^13^ Department of Gastroenterology and Hepatology Yokohama City University School of Medicine Yokohama Japan; ^14^ Department of Hepatology The Affiliated Hospital of Hangzhou Normal University Hangzhou China; ^15^ The Third Unit Department of Hepatology Beijing Youan Hospital Capital Medical University Beijing China; ^16^ Gastroenterology and Hepatology Unit Department of Medicine Faculty of Medicine University of Malaya Kuala Lumpur Malaysia; ^17^ Department of Hepatobiliary Pancreatic Surgery Eighth Hospital Affiliated to Sun Yat‐Sen University Shenzhen Guangdong Province China; ^18^ Institute For the Study of Liver Diseases The Third People's Hospital of Changzhou Changzhou Jiangsu Province China; ^19^ Department of Hepatology Hepatology Research Institute The First Affiliated Hospital of Fujian Medical University Fuzhou China; ^20^ Centre D'investigation De La Fibrose Hépatique Hôpital Haut‐Lévêque Bordeaux University Hospital Pessac France; ^21^ Department of Medical Imaging “Prof. Dr. Octavian Fodor” Regional Institute of Gastroenterology and Hepatology ‘’Iuliu Hațieganu” University of Medicine and Pharmacy Cluj‐Napoca Romania; ^22^ Clinical Laboratory Center The Fifth People's Hospital of Wuxi Wuxi Jiangsu China; ^23^ Center of Infectious Diseases West China Hospital Sichuan University Chengdu China; ^24^ Liver Research Center Beijing Friendship Hospital Capital Medical University Beijing China; ^25^ National Institute for Health Research Nottingham Biomedical Research Centre Nottingham University Hospitals NHS Trust and University of Nottingham Nottingham UK; ^26^ Department of Hepatology Tianjin Second People's Hospital Tianjin China; ^27^ Department of Gastroenterology First Affiliated Hospital of China Medical University Shenyang China; ^28^ Department of Traditional and Western Medical Hepatology The Third Hospital of Hebei Medical University Shijiazhuang China; ^29^ Department of Infectious Diseases The Third Affiliated Hospital of Sun Yat‐Sen University Guangzhou China; ^30^ Department of Hepatology Center of Infectious Diseases and Pathogen Biology The First Hospital of Jilin University Changchun China; ^31^ Department of Infectious Disease The Affiliated Hospital of Xuzhou Medical University Xuzhou Jiangsu China; ^32^ Department of Hepatology of The Third Central Hospital of Tianjin Tianjin China; ^33^ Department of Infectious Diseases The First Affiliated Hospital of Zhengzhou University Zhengzhou China; ^34^ Department of Gastroenterology and Hepatology Guangzhou First People's Hospital School of Medicine South China University of Technology Guangzhou China; ^35^ Translational Gastroenterology Unit University of Oxford Oxford UK; ^36^ Institute of Liver Diseases Shuguang Hospital Affiliated to Shanghai University of Traditional Chinese Medicine Shanghai China; ^37^ Department of Infectious Disease Nanjing Drum Tower Hospital The Affiliated Hospital of Nanjing University Medical School Nanjing Jiangsu China; ^38^ Department of Infectious Diseases The First Affiliated Hospital of Anhui Medical University Hefei China; ^39^ Department of Hepatobiliary Diseases Affiliated Traditional Chinese Medicine Hospital of Southwest Medical University Chengdu Sichuan Province China; ^40^ Department of Infectious Diseases The Second Affiliated Hospital of Xi'an Jiaotong University Xi'an China; ^41^ Department of Gastroenterology Southwest Hospital Army Medical University Chongqing China; ^42^ Department of Infectious Diseases Qingdao Municipal Hospital Affiliated to Qingdao University Qingdao China; ^43^ Department of Medicine University of Verona Verona Italy; ^44^ IRCCS Sacro Cuore‐Don Calabria Hospital Negrar di Valpolicella Italy; ^45^ Southampton National Institute For Health and Care Research Biomedical Research Centre University Hospital Southampton and University of Southampton Southampton General Hospital Southampton UK; ^46^ Department of Hepatology Qilu Hospital of Shandong University Jinan China; ^47^ Department of Paediatrics The Affiliated Wuxi Children's Hospital of Jiangnan University Wuxi Jiangsu China; ^48^ Centre For Obesity Research and Education Department of Surgery Monash University Melbourne Australia; ^49^ Storr Liver Centre Westmead Institute for Medical Research Westmead Hospital and University of Sydney Sydney NSW Australia; ^50^ Oxford Centre for Clinical Magnetic Resonance Research Radcliffe Department of Medicine and Oxford NIHR Biomedical Research Centre University of Oxford Oxford UK; ^51^ MAFLD Research Center Department of Hepatology the First Affiliated Hospital of Wenzhou Medical University Wenzhou China; ^52^ Key Laboratory of Diagnosis and Treatment for The Development of Chronic Liver Disease in Zhejiang Province Wenzhou Zhejiang China

**Keywords:** epidemiology, estimated glomerular filtration rate, liver fibrosis, metabolic dysfunction‐associated steatotic liver disease, metabolic dysfunction‐associated fatty liver disease

## Abstract

Metabolic dysfunction‐associated steatotic liver disease (MASLD) has become the most prevalent chronic liver disease globally. Previous studies have shown that MASLD is an independent risk factor for chronic kidney disease (CKD), but the variations in estimated glomerular filtration rate (eGFR) levels across countries with different ethnic backgrounds have not been extensively reported. We enrolled 3308 participants with biopsy‐proven MASLD from 34 centers in this multinational study and analyzed the associations between eGFR and histological severity of liver fibrosis in different countries. European participants had lower eGFR levels (92.2 ± 20.7 vs. 104.7 ± 17.3 mL/min/1.73 m^2^) and significant liver fibrosis (61.4 vs. 32.4%) than Asian individuals. In Asia, Chinese participants had the highest mean eGFR level at 105.8 mL/min/1.73 m^2^, while Malaysian participants had the lowest at 87.3 mL/min/1.73 m^2^ (*p* < 0.001). In Europe, French participants had the highest mean eGFR level at 95.3 mL/min/1.73 m^2^, while Romanian individuals had the lowest at 81.1 mL/min/1.73 m^2^ (*p* < 0.001). eGFR levels were inversely associated with liver fibrosis in Asian individuals (OR: 0.793, 95%CI: 0.685–0.917, *p* = 0.002), even after adjusting for traditional renal risk factors, but not in Europeans. Our findings provide the basis for further investigation of the burden of MASLD on CKD risk in different countries.

## Graphical abstract

A total of 3308 participants with biopsy‐proven MASLD from 34 centers were enrolled in the study. We collected liver biopsy pathological scores and clinical parameters and conducted correlation analysis. We finally found that eGFR levels are inversely associated with liver fibrosis in Asians, even after adjusting for traditional renal risk factors.

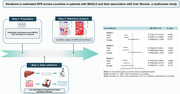

## 1 Introduction

Metabolic dysfunction‐associated steatotic liver disease (MASLD) has become a significant public health problem with the increasing global epidemic of obesity, resulting in substantial social and economic burdens in many global regions [1, 2, 3]. Similar to the metabolic dysfunction‐associated fatty liver disease (MAFLD) definition, this newly proposed nomenclature better emphasizes the pathogenic role of metabolic dysfunction in this common steatotic liver disease [4, 5, 6, 7]. It has also been reported that MASLD may contribute to the development of extra‐hepatic complications, such as cardiovascular disease and chronic kidney disease (CKD) [8, 9, 10, 11, 12].

CKD is a major public health problem worldwide, with a global prevalence of more than 10% that is increasing year by year [13, 14]. Previous studies have shown that the prevalence of CKD is greater in individuals with MASLD and liver fibrosis compared with their counterparts with no liver fibrosis [15, 16, 17, 18], and that the incidence of CKD with or without proteinuria is independently associated with liver fibrosis, even after adjusting for traditional renal risk factors [15]. We have systematically described the potential mechanisms involved in the link between MASLD and CKD [9]. In addition, international experts from 26 countries contributed to a consensus on the relationship between MASLD and CKD through a Delphi investigation, guiding the prevention and treatment of both MASLD and CKD [19].

Estimated glomerular filtration rate (eGFR) is a key indicator for assessing renal function, which is crucial for early detection and assessment of CKD, guiding medication use and predicting prognosis [20]. eGFR decline may suggest severe kidney damage and is closely associated with the risk of end‐stage renal disease and mortality [21]. eGFR decline is also closely related to the progression of various extra‐renal diseases, including cardiovascular disease and certain cancers [22, 23]. Moreover, our previous study analyzed and predicted the relationship between liver fibrosis and eGFR using artificial intelligence in MASLD [24]. However, this relationship needs to be further verified in multicenter cohort studies and the geographical variations of eGFR across countries among patients with MASLD need to be further explored.

In this multinational cohort study, we combined data from 34 centers across European and Asian countries to illustrate the epidemiological distribution of eGFR levels among individuals with biopsy‐confirmed MASLD. We examined the association between eGFR level and the histological severity of liver fibrosis, aiming to provide new insights into the prevention of CKD in people with MASLD.

## 2 Results

### 2.1 Clinical Characteristics of MASLD Population

**FIGURE 1:**
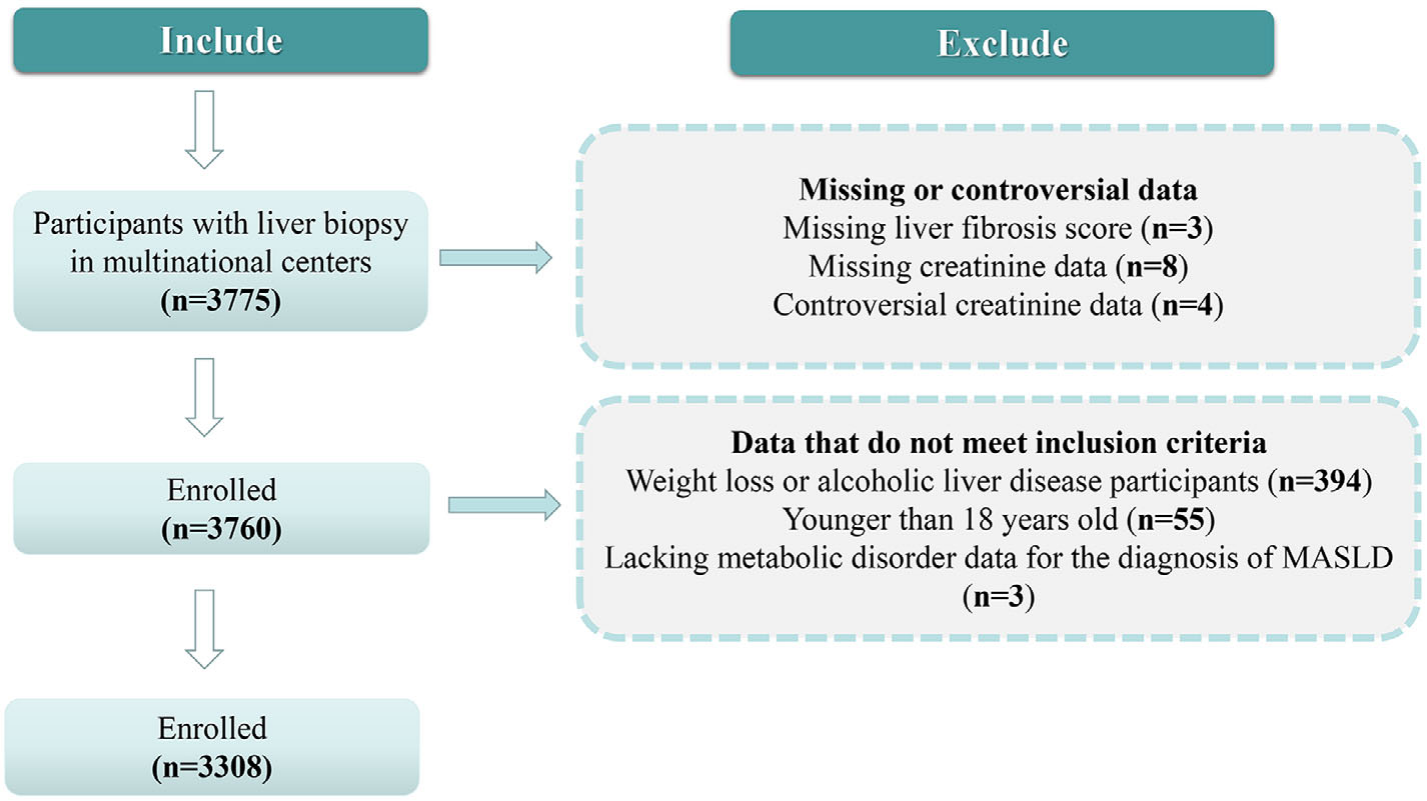
The flow chart of MASLD population enrollment. MASLD, metabolic dysfunction‐associated steatotic liver disease.

As shown in Figure 1, in this multinational study, we enrolled 3308 adult participants with biopsy‐confirmed MASLD. Table 1 shows the main clinical and biochemical characteristics, as well as the liver biopsy features of MASLD participants. The average age of participants was 43 ± 14 years, and the proportion of men was 59.0%.

**TABLE 1:** Characteristics of MASLD participants stratified by region and country.

	Asia					Europe				
Characteristics	Total (*n* = 3308)	Total (*n* = 2982)	China (*n* = 2628)	Malaysia (*n* = 79)	Japan (*n* = 275)	Total (*n* = 326)	France (*n* = 68)	Germany (*n* = 120)	Romania (*n* = 60)	UK (*n* = 78)
**Clinical parameters**										
Age (years)	43 ± 14	42 ± 14	41 ± 13	52 ± 12	56 ± 14	50 ± 14^a^	57 ± 13	48 ± 13	42 ± 11	52 ± 13
Male sex (*n*, %)	1950 (59.0)	1751 (58.7)	1585 (60.3)	35 (44.3)	131 (47.6)	199 (61.0)	41 (60.3)	68 (56.7)	43 (71.7)	47 (60.3)
BMI (kg/m^2^)	27.6 ± 4.6	27.1 ± 4.3	27.1 ± 4.3	28.7 ± 4.8	26.9 ± 3.9	31.5 ± 5.5^a^	30.4 ± 5.4	31.6 ± 5.4	29.7 ± 4.1	33.6 ± 6.0
Waist circumference (cm)	95.3 ± 12.3	93.9 ± 11.3	93.8 ± 11.5	93.3 ± 11.1	95.1 ± 9.2	106.5 ± 13.8^a^	104.7 ± 14.6	NA	102.2 ± 13.0	110.8 ± 12.3
Platelet count (×10^9^/L)	230.5 ± 66.0	231.5 ± 65.8	231.9 ± 64.5	269.4 ± 70.5	216.4 ± 71.7	221.6 ± 67.1^a^	185.4 ± 68.9	237.7 ± 62.6	245.1 ± 49.6	210.5 ± 69.2
Glucose (mmol/L)	6.0 ± 2.1	5.9 ± 2.0	5.9 ± 2.1	6.4 ± 1.9	6.1 ± 1.7	6.7 ± 2.7^a^	6.9 ± 2.9	NA	6.2 ± 1.9	7.0 ± 3.0
AST (U/L)	52.8 ± 36.4	52.6 ± 36.7	52.6 ± 36.9	43.2 ± 33.6	55.1 ± 34.6	54.8 ± 33.9	62.4 ± 38.2	59.3 ± 34.4	48.5 ± 35.6	46.0 ± 24.2
ALT (U/L)	85.8 ± 65.9	86.2 ± 66.4	87.5 ± 67.6	71.0 ± 50.2	79.4 ± 57.5	81.7 ± 61.4	75.4 ± 52.6	90.9 ± 78.2	93.6 ± 52.4	63.8 ± 37.2
Creatinine (µmol/L)	70.5 ± 17.3	69.7 ± 16.6	69.9 ± 15.6	80.4 ± 25.8	64.2 ± 19.8	78.3 ± 21.4^a^	70.9 ± 26.5	76.4 ± 13.2	96.6 ± 22.3	73.6 ± 18.1
eGFR (mL/min/1.73 m^2^)	103.5 ± 18.1	104.7 ± 17.3	105.8 ± 16.8	87.3 ± 22.0	99.4 ± 17.5	92.2 ± 20.7^a^	95.3 ± 21.4	94.3 ± 15.9	81.1 ± 24.8	94.9 ± 20.6
Declined eGFR (%)	681 (20.6)	536 (18.0)	429 (16.3)	43 (54.4)	64 (23.3)	145 (44.5)^a^	22 (32.4)	51 (42.5)	44 (73.3)	28 (35.9)
**Metabolic comorbidities**										
Obesity (%)	1262 (38.2)	1082 (36.3)	934 (35.5)	41 (51.9)	107 (38.9)	180 (55.2)^a^	33 (48.5)	67 (55.8)	23 (38.3)	57 (73.1)
Type 2 diabetes (%)	778 (23.5)	700 (23.5)	513 (19.5)	45 (57.0)	142 (51.6)	78 (53.8)^a^	37 (54.4)	NA	NA	41 (52.6)
Hypertension (%)	793 (24.0)	735 (24.7)	547 (20.8)	46 (58.2)	142 (51.6)	58 (49.2)^a^	38 (55.9)	NA	NA	20 (25.6)
**Liver biopsy features**										
Steatosis score										
0	45 (1.4)	36 (1.2)	35 (1.3)	0 (0.0)	1 (0.4)	9 (2.8)^a^	4 (5.9)	5 (4.2)	0 (0.0)	0 (0.0)
1	1103 (33.3)	988 (33.1)	795 (30.3)	31 (39.2)	162 (58.9)	115 (35.2)	22 (32.3)	46 (38.3)	22 (36.7)	25 (32.0)
2	1269 (38.4)	1142 (38.3)	1033 (39.3)	26 (33.0)	83 (30.2)	127 (39.0)	20 (29.4)	62 (51.7)	21 (35.0)	24 (30.8)
3	891 (26.9)	816 (27.4)	765 (29.1)	22 (27.8)	29 (10.5)	75 (23.0)	22 (32.4)	7 (5.8)	17 (28.3)	29 (37.2)
Significant steatosis (*n*, %)	2160 (65.3)	1958 (65.7)	1798 (68.4)	48 (60.8)	112 (40.7)	202 (62.0)	42 (61.8)	69 (57.5)	38 (63.3)	53 (68.0)
Inflammation score										
0	174 (5.3)	118 (4.0)	77 (2.9)	2 (2.5)	39 (14.2)	56 (17.2)^a^	10 (14.7)	29 (24.2)	1 (1.7)	16 (20.5)
1	1783 (53.9)	1600 (53.6)	1407 (53.6)	41 (51.9)	152 (55.3)	184 (56.4)	45 (66.2)	70 (58.3)	24 (40.0)	45 (57.7)
2	1203 (36.4)	1133 (38.0)	1025 (39.0)	35 (44.3)	73 (26.5)	70 (21.5)^a^	12 (17.6)	20 (16.7)	22 (36.7)	16 (20.5)
3	147 (4.4)	131 (4.4)	119 (4.5)	1 (1.3)	11 (4.0)	16 (4.9)	1 (1.5)	1 (0.8)	13 (21.6)	1 (1.3)
Significant inflammation (*n*, %)	1350 (40.8)	1264 (42.4)	1144 (43.5)	36 (45.6)	84 (30.5)	86 (26.4)^a^	13 (19.1)	21 (17.5)	35 (58.3)	17 (21.8)
Ballooning score										
0	513 (15.5)	440 (14.8)	334 (12.7)	7 (8.9)	99 (36.0)	73 (22.4)^a^	19 (27.9)	35 (29.2)	3 (5.0)	16 (20.5)
1	1617 (48.9)	1459 (48.9)	1314 (50.0)	42 (53.1)	103 (37.4)	158 (48.5)	28 (41.2)	71 (59.2)	24 (50.0)	35 (44.9)
2	1178 (35.6)	1083 (36.3)	980 (37.3)	30 (38.0)	73 (26.6)	95 (29.1)^a^	21 (30.9)	14 (11.6)	33 (55.0)	27 (34.6)
Significant ballooning (*n*, %)	1178 (35.6)	1083 (36.3)	980 (37.3)	30 (38.0)	73 (26.6)	95 (29.1)^a^	21 (30.9)	14 (11.6)	33 (55.0)	27 (34.6)
Fibrosis score										
0	535 (16.2)	497 (16.7)	405 (15.4)	2 (2.5)	90 (32.7)	38 (11.6)^a^	6 (8.8)	7 (5.8)	18 (30.0)	7 (9.0)
1	1605 (48.5)	1517 (50.9)	1415 (53.8)	31 (39.3)	71 (25.8)	88 (27.0)^a^	9 (13.2)	35 (29.2)	26 (43.3)	18 (23.1)
2	714 (21.6)	617 (20.7)	544 (20.7)	27 (34.2)	46 (16.7)	97 (29.8)^a^	13 (19.1)	57 (47.5)	12 (20.0)	15 (19.2)
3	315 (9.5)	246 (8.2)	180 (6.9)	14 (17.7)	52 (19.0)	69 (21.2)^a^	15 (22.1)	21 (17.5)	4 (6.7)	29 (37.2)
4	139 (4.2)	105 (3.5)	84 (3.2)	5 (6.3)	16 (5.8)	34 (10.4)^a^	25 (36.8)	0 (0.0)	0 (0.0)	9 (11.6)
Significant fibrosis (*n*, %)	1168 (35.3)	968 (32.4)	808 (30.8)	46 (58.2)	114 (41.5)	200 (61.4)^a^	53 (78.0)	78 (65.0)	16 (26.7)	53 (68.0)

Abbreviations: MASLD = metabolic dysfunction‐associated steatotic liver disease, AST = aspartate aminotransferase, ALT = alanine aminotransferase, BMI = body mass index, eGFR = estimated glomerular filtration rate, NA = not available. Declined eGFR was defined as eGFR < 90 mL/min/1.73 m^2^. Data were expressed as means ± standard deviation (SD).

^a^ *p* < 0.05 or less for comparison between Asian and European populations.

The prevalence of type 2 diabetes and hypertension was 23.5 and 24.0%, respectively. The prevalence of obesity was 38.2%, and the mean body mass index (BMI) was 27.6 ± 4.6 kg/m^2^. The mean eGFR level (as estimated by the CKD‐EPI equation) of the total population was 103.5 ± 18.1 mL/min/1.73 m^2^, and 20.6% had a decreased eGFR (defined as eGFR < 90 mL/min/1.73 m^2^). For liver biopsy data, the proportion of significant steatosis, inflammation, ballooning, and fibrosis were 65.3, 40.8, 35.6, and 35.3%, respectively.

### 2.2 Clinical Characteristics of MASLD in Different Regions

As shown in Table 1, European participants (*n* = 326) were more likely to be older, and had significantly higher fasting glucose levels and a greater prevalence of type 2 diabetes and hypertension than Asian participants (*n* = 2982). European participants also had a significantly higher serum creatinine (78.3 ± 21.4 vs. 69.7 ± 16.6 µmol/L), lower eGFR (92.2 ± 20.7 vs. 104.7 ± 17.3 mL/min/1.73 m^2^) and a greater prevalence of significant fibrosis (61.4 vs. 32.4%) than Asian participants. As shown in Table 2, in Asia and Europe, participants with declined eGFR were older than those with normal eGFR. In Asian participants with declined eGFR, there was a higher prevalence of diabetes and hypertension and lower levels of platelets and alanine aminotransferase (ALT) concentration; this difference was not observed in the European population. Furthermore, Asian participants with declined eGFR had significantly higher proportion of significant liver fibrosis, while European participants with declined eGFR had more significant inflammation.

**TABLE 2:** Characteristics of MASLD participants stratified by eGFR values in Asian and European populations.

	Asian (*n* = 2982)			European (*n* = 326)		
Characteristics	eGFR < 90 mL/min/1.73 m^2^ (*n* = 536)	eGFR ≥ 90 mL/min/1.73 m^2^ (*n* = 2446)	*p* Value	eGFR < 90 mL/min/1.73 m^2^ (*n* = 145)	eGFR ≥ 90 mL/min/1.73 m^2^ (*n* = 181)	*p* Value
**Clinical parameters**						
Age (years)	51.0 ± 14.2	40.4 ± 13.3	<0.001	54.9 ± 12.5	45.1 ± 13.0	<0.001
Male sex (*n*, %)	199 (37.1)	1552 (63.5)	<0.001	78 (53.8)	121 (66.9)	0.016
BMI (kg/m^2^)	26.8 ± 4.0	27.2 ± 4.3	0.059	31.5 ± 5.5	31.5 ± 5.5	0.998
Waist circumference (cm)	91.5 ± 10.9	94.4 ± 11.4	<0.001	106.3 ± 12.8	106.5 ± 14.5	0.928
Platelet count (×10^9^/L)	221.8 ± 67.3	233.6 ± 65.3	<0.001	226.1 ± 66.1	218.0 ± 67.9	0.280
Glucose (mmol/L)	6.0 ± 1.8	5.9 ± 2.1	0.733	6.7 ± 2.4	6.8 ± 3.0	0.818
AST (U/L)	49.3 ± 35.0	53.3 ± 37.0	0.021	52.1 ± 32.0	56.9 ± 35.2	0.198
ALT (U/L)	71.6 ± 58.4	89.5 ± 67.6	<0.001	77.0 ± 48.9	85.4 ± 69.8	0.218
Creatinine (µmol/L)	85.5 ± 17.9	66.2 ± 14.0	<0.001	91.7 ± 22.0	67.5 ± 13.2	<0.001
eGFR (mL/min/1.73m^2^)	78.1 ± 11.1	110.5 ± 12.2	<0.001	72.8 ± 14.2	105.8 ± 12.3	0.000
**Metabolic comorbidities**						
Obesity (%)	165 (30.8)	917 (37.5)	0.003	80 (55.2)	100 (55.3)	0.989
Type 2 diabetes (%)	162 (30.2)	538 (22.0)	<0.001	29 (20.0)	49 (27.1)	0.461
Hypertension (%)	191 (35.6)	544 (22.2)	<0.001	25 (17.2)	33 (18.2)	0.061
**Liver biopsy features**						
Steatosis score						
0	6 (1.1)	30 (1.2)	0.837	5 (3.4)	4 (2.2)	0.498
1	209 (39.0)	779 (31.8)	0.001	49 (33.8)	66 (35.5)	0.616
2	187 (34.9)	955 (39.0)	0.073	62 (42.8)	65 (35.9)	0.208
3	134 (25.0)	682 (27.9)	0.175	29 (20.0)	46 (25.4)	0.248
Significant steatosis (*n*, %)	321 (59.9)	1637 (66.9)	0.002	91 (62.8)	111 (61.3)	0.791
Inflammation score						
0	28 (5.2)	90 (3.7)	0.097	19 (13.1)	37 (20.4)	0.081
1	273 (50.9)	1327 (54.3)	0.163	76 (52.4)	108 (59.7)	0.189
2	210 (39.2)	923 (37.8)	0.533	39 (26.9)	31 (17.1)	0.033
3	25 (4.6)	106 (4.3)	0.735	11 (7.6)	5 (2.8)	0.045
Significant inflammation (*n*, %)	235 (43.8)	1029 (42.1)	0.451	50 (34.5)	36 (19.9)	0.003
Ballooning score						
0	73 (13.6)	367 (15.0)	0.413	25 (17.3)	48 (26.5)	0.046
1	287 (53.6)	1172 (47.9)	0.018	73 (50.3)	85 (47.0)	0.544
2	176 (32.8)	907 (37.1)	0.064	47 (32.4)	48 (26.5)	0.244
Significant ballooning (*n*, %)	176 (32.8)	907 (37.1)	0.064	47 (32.4)	48 (26.5)	0.244
Fibrosis score						
0	75 (14.0)	422 (17.3)	0.067	21 (14.5)	17 (9.4)	0.155
1	240 (44.8)	1277 (52.2)	0.002	36 (24.8)	52 (28.7)	0.430
2	130 (24.2)	487 (19.9)	0.025	41 (28.3)	56 (30.9)	0.601
3	59 (11.0)	187 (7.6)	0.010	31 (21.4)	38 (21.0)	0.933
4	32 (6.0)	73 (3.0)	0.001	16 (11.0)	18 (10.0)	0.749
Significant fibrosis (*n*, %)	221 (41.2)	747 (30.5)	<0.001	88 (60.7)	112 (61.9)	0.827

Data were expressed as means ± standard deviation (SD).

Abbreviations: MASLD = metabolic dysfunction‐associated steatotic liver disease, AST = aspartate aminotransferase, ALT = alanine aminotransferase, BMI = body mass index, eGFR = estimated glomerular filtration rate.

### 2.3 Clinical Characteristics of MASLD Population in Different Countries

**FIGURE 2:**
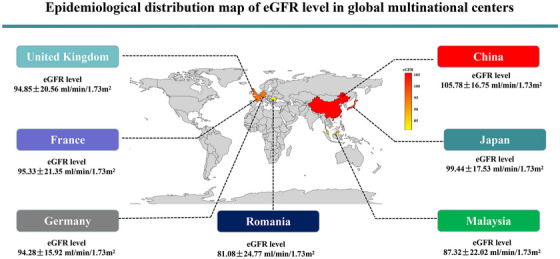
Epidemiological distribution map of eGFR level in global multinational centers. The epidemiological distribution map was drawn according to the Standard map service of Ministry of Natural Resources of the People's Republic of China. Map approval number: GS(2016)1553. eGFR, estimated glomerular filtration rate.

MASLD data in each country were analyzed individually. In Table 1 and Figure 2, data show that Malaysia had the highest prevalence of diabetes and hypertension, 57.0 and 58.2%, respectively. The UK had the highest prevalence of obesity at 73.1% with a mean (SD) eGFR level of 94.9 ± 20.6 mL/min/1.73 m^2^. In Europe, France had the highest mean eGFR level at 95.3 mL/min/1.73 m^2^ and Romania had the lowest at 81.1 mL/min/1.73 m^2^. In Asia, China had the highest mean eGFR level at 105.8 mL/min/1.73 m^2^ and Malaysia had the lowest at 87.3 mL/min/1.73 m^2^. Malaysia had the highest proportion of significant liver fibrosis at 58.2%, and China had the lowest at 30.8%. France had the highest proportion of significant liver fibrosis at 78.0% and Romania had the lowest at 26.7%.

### 2.4 Correlation Analysis Between eGFR and Liver Fibrosis in Asia and Europe

**FIGURE 3:**
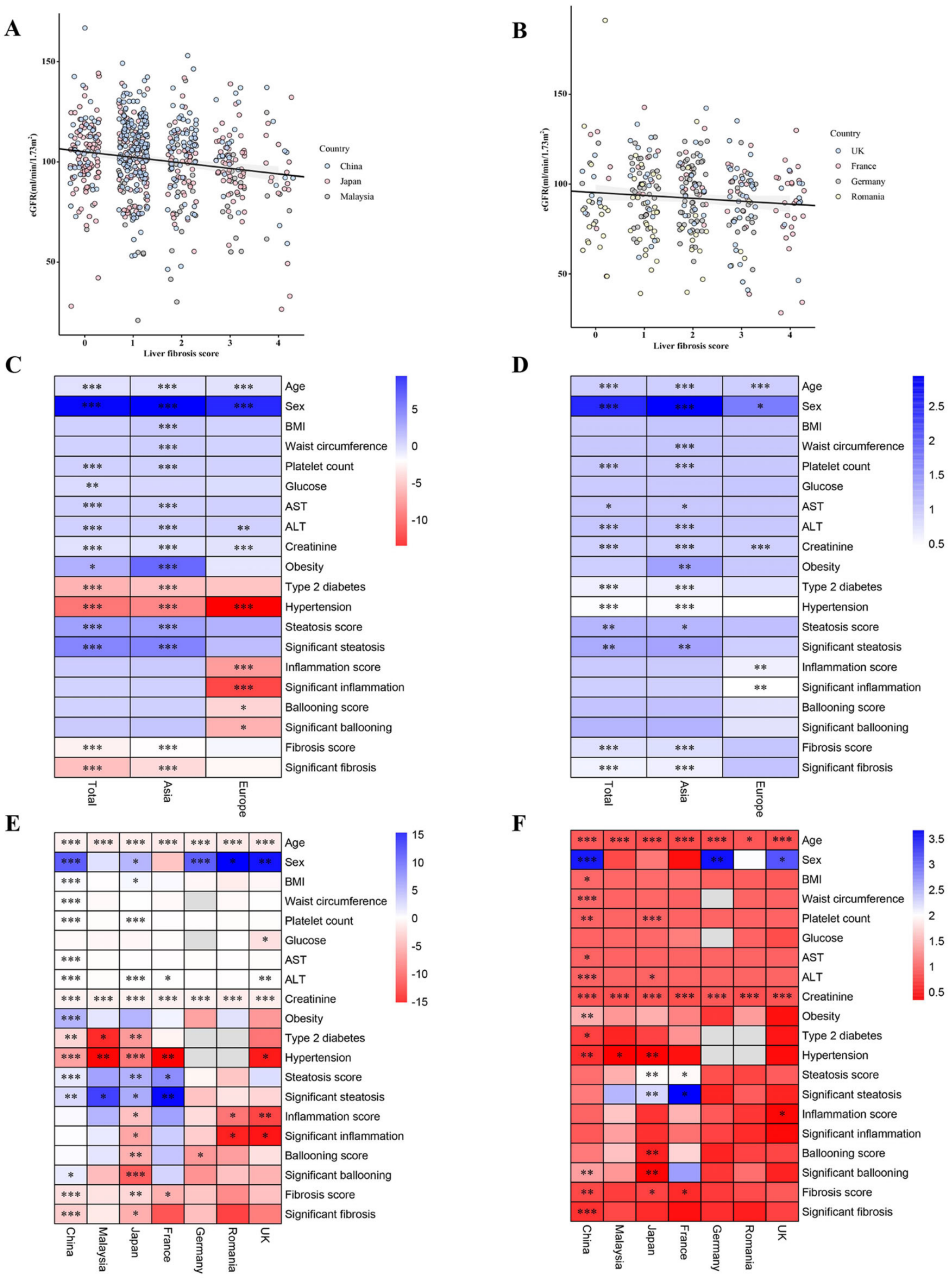
Trend chart and association between eGFR and liver fibrosis score in different regions. In the scatter plot, each point represents the liver fibrosis score and eGFR values for a specific individual. To reduce data redundancy and improve visualization, a random sample of 300 data points was taken for China, which had a larger sample size. Jitter was added to the plot to alleviate point overlap and enhance clarity. Additionally, a linear curve and its corresponding 95% confidence interval were included to illustrate the overall trend between liver fibrosis score and eGFR. (A) Trend chart between eGFR and liver fibrosis score in Asia. (B) Trend chart between eGFR and liver fibrosis score in Europe. (C) Heat map of linear regression analysis between eGFR and liver fibrosis score in different regions, where different colors indicated the *β* value. ***p < 0.001, **p < 0.01, *p < 0.05. (D) Heat map of binary logistic regression analysis between eGFR and liver fibrosis score in different regions, where different colors indicated the OR value. ***p < 0.001, **p < 0.01, *p < 0.05. (E) Heat map of linear regression analysis between eGFR and liver fibrosis score in different countries, where different colors indicated the *β* value. ***p < 0.001, **p < 0.01, *p < 0.05. (F) Heat map of binary logistic regression analysis between eGFR and liver fibrosis score in different countries, where different colors indicated the OR value. ***p < 0.001, **p < 0.01, *p < 0.05. AST, aspartate aminotransferase; ALT, alanine aminotransferase; BMI, body mass index; eGFR, estimated glomerular filtration rate.

**FIGURE 4:**
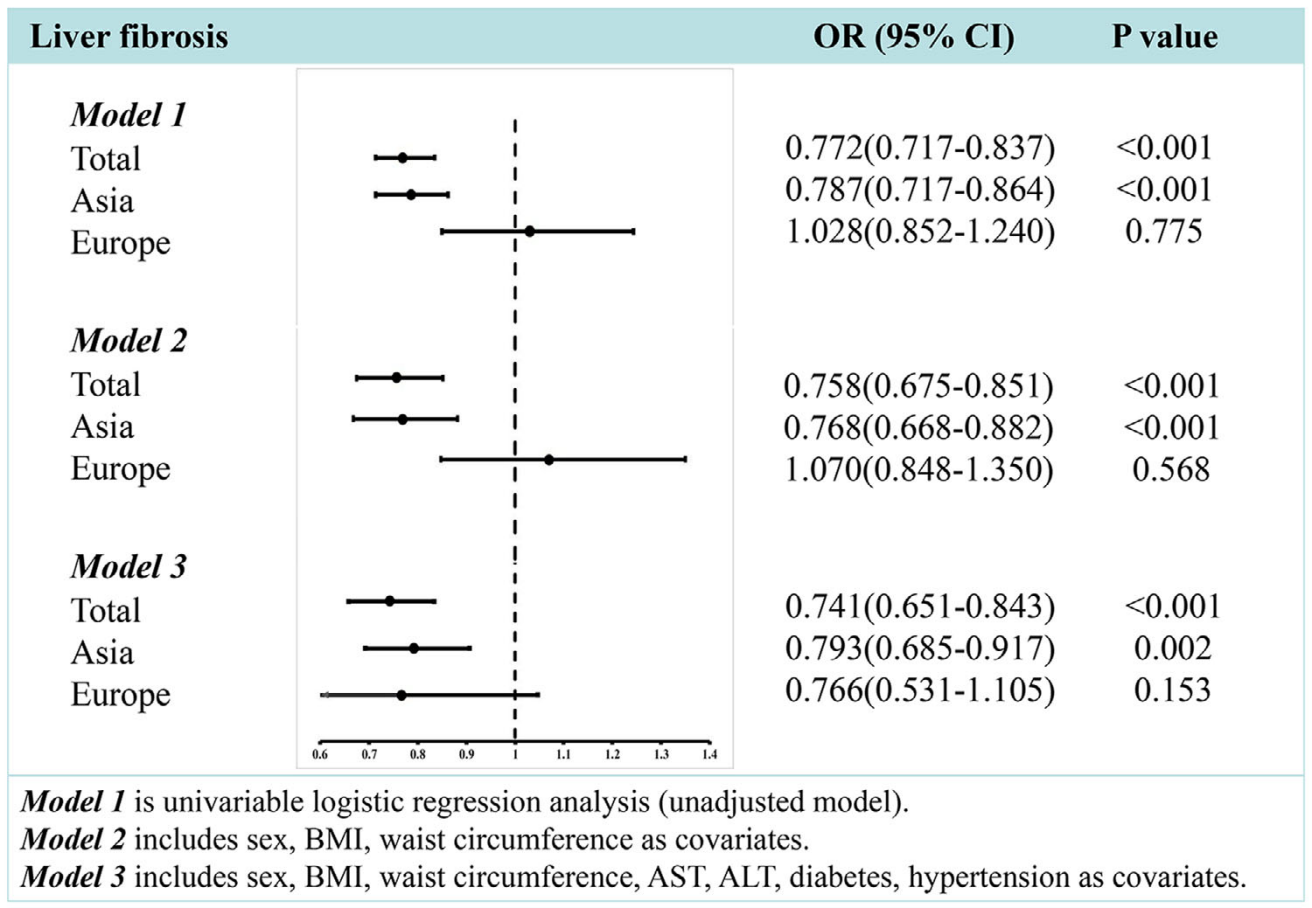
Association between eGFR and liver fibrosis score in different regions after adjusting for traditional metabolic factors. AST, aspartate aminotransferase; ALT, alanine aminotransferase; BMI, body mass index; eGFR, estimated glomerular filtration rate.

As reported in Figure 3A,B, eGFR levels decreased gradually with the increase in liver fibrosis scores both in Asia and Europe. In Figure 3C,D, the eGFR level was inversely associated with liver fibrosis score (*β*: −2.088, 95% confidence interval [CI]: −2.730, −1.445, *p* < 0.001) in Asia. However, this association was not significant in Europe. In univariable linear regression analyses, as shown in Figure 3E,F, eGFR level was inversely associated with liver fibrosis score in China (*β*: −1.679, 95% CI: −2.375, −0.984, *p* < 0.001), Japan (*β*: −2.214, 95% CI: −3.830, −0.596, *p* = 0.007) and France (*β*: −4.483, 95% CI: −8.253, −0.713, *p* = 0.021), but not in Malaysia, Germany, Romania, and the UK. In binary logistic regression analyses, there was a significant inverse association between eGFR and liver fibrosis score in China (odds ratio [OR] 0.827, 95% CI: 0.742–0.921), Japan (OR 0.793, 95% CI: 0.638–0.985), and France (OR 0.628, 95% CI: 0.402–0.982), but not in Malaysia, Germany, Romania, and the UK. In addition, there was a significant inverse association between eGFR and liver fibrosis score (OR: 0.787, 95% CI: 0.717–0.864) in the pooled Asian population. After adjusting for traditional renal risk factors, such as sex, adiposity measures (BMI, waist circumference), hypertension, diabetes, and serum transaminases, eGFR remained significantly associated with liver fibrosis in the Asian population (adjusted OR 0.793, 95% CI: 0.685–0.917) (see Figure 4 and Table 3) but not in the European population. These results suggest that the histological severity of liver fibrosis in MASLD increased significantly with decreasing eGFR levels in Asian but not European participants.

**TABLE 3:** Logistic regression analysis based on eGFR in different populations.

	Model 1		Model 2		Model 3	
	OR (95% CI)	*p*	OR (95% CI)	*p*	OR (95% CI)	*p*
Non‐type 2 diabetes	0.734 (0.657–0.819)	<0.001	0.706 (0.597–0.836)	<0.001	0.712 (0.598–0.849)	<0.001
Type 2 diabetes	0.881 (0.766–1.013)	0.076	0.774 (0.646–0.927)	0.005	0.743 (0.609–0.908)	0.004
Non‐hypertension	0.735 (0.658–0.822)	<0.001	0.708 (0.598–0.839)	<0.001	0.693 (0.579–0.831)	<0.001
Hypertension	0.874 (0.761–1.002)	0.054	0.780 (0.653–0.932)	0.006	0.795 (0.659–0.960)	0.017
Non‐obesity	0.775 (0.705–0.851)	<0.001	0.756 (0.660–0.866)	<0.001	0.754 (0.649–0.876)	<0.001
Obesity	0.762 (0.645–0.899)	0.001	0.754 (0.598–0.950)	0.017	0.698 (0.534–0.912)	0.008

Model 1 is an univariable logistic regression analysis (unadjusted model).

Model 2 was adjusted for sex, BMI, and waist circumference as covariates (except for stratified analyses by obesity status, which were adjusted only for sex).

Model 3 was adjusted for sex, BMI, waist circumference, diabetes, hypertension, AST, and ALT as covariates (adiposity measures, diabetes, or hypertension were included as covariates only in appropriate stratified analyses).

## 3 Discussion

To our knowledge, this multinational cohort study is the first to systematically describe the geographical variations of eGFR levels in adult individuals with biopsy‐confirmed MASLD across different countries and to analyze the associations between eGFR levels and the histological severity of liver fibrosis. Our results serve as a call to physicians to pay more attention to assessing the risk of CKD in individuals with MASLD. Importantly, our data show that there are significant differences between countries and regions in the association between eGFR levels and liver fibrosis severity, with an independent inverse association observed only in Asian countries and not in European countries.

A large meta‐analysis of more than 10 million individuals reported that the global prevalence of MASLD was 38.8% (95% CI 32.9–44.9%) [25], reaching 50.7% (95% CI 46.9–54.4%) in the overweight and obesity populations [26], thus further highlighting that MASLD has become a serious public health problem worldwide. Growing evidence showed that individuals with MASLD are at high risk of developing CKD [14, 15, 27].

Currently, there are a lack of large epidemiological studies describing the geographical distribution of eGFR levels among adult individuals with MASLD in different countries worldwide. After pooling individual data of 3308 individuals with biopsy‐proven MASLD from 34 centers in seven countries, we compared the clinical data between Asian and European participants and found that European people had significantly higher adiposity measures and a greater prevalence of metabolic syndrome traits involved in the diagnosis of MASLD, than Asian individuals. Europeans with MASLD also had lower eGFR levels than Asians. Further analysis in each country showed that China had the highest eGFR levels and Malaysia had the lowest amongst Asian countries, whilst France had the highest eGFR levels and Romania had the lowest eGFR levels in Europe. This is partly consistent with the results from a previous study showing that although the overall prevalence of CKD in China was lower than in Japan and Malaysia, China is becoming the country with the largest number of individuals with CKD because of its large overall population [13]. We also found that amongst the European countries, the eGFR level was lower in Romania than in France, Germany, and UK, but no relevant research exists to explain this difference. Brück et al. [28] reported that the eGFR level in Europe varied from 80.7 mL/min/1.73 m^2^ in Ireland to 100.4 mL/min/1.73 m^2^ in Italy, but this study did not include data from Germany and UK. Therefore, this finding requires further exploration.

When we stratified our participants with MASLD into those with a normal eGFR level (i.e., ≥90 mL/min/1.73 m^2^, which is the most commonly used cutoff value for defining normal renal function [29, 30]) and those with a declined eGFR level (<90 mL/min/1.73 m^2^), we found that both in Asia and Europe, participants with a declined eGFR were older and more likely to be female, suggesting that we should pay more attention to the risk of developing CKD in the elderly female MASLD population. Moreover, European participants with a decreased eGFR also had a higher prevalence of type 2 diabetes and hypertension and lower platelet counts than their counterparts with a normal eGFR. Although obesity has commonly been recognized as a risk factor for CKD [31, 32], in our study, the proportion of obese population with eGFR below 90 mL/min/1.73 m^2^ was significantly decreased. A possible explanation for this finding might be due to the abnormal hemodynamics in obese individuals during early stages of renal dysfunction, thereby leading to an increase in glomerular perfusion and filtration pressure and a subsequent eGFR reduction [33].

Liver biopsy remains the reference standard for diagnosing MASLD, and liver histology scores play an important role in assessing the severity of liver disease [34]. Among these liver histology scores, the severity of liver fibrosis has been reported to be closely related to renal function [15, 19, 35]. Notably, our multinational cohort study found that participants with decreased eGFR had higher liver fibrosis scores and a greater proportion of significant liver fibrosis (score F2 or greater), especially in the Asian population, consistent with previous studies [7, 15, 19]. However, this phenomenon seems to be different in the European population since we found that participants with declined eGFR had higher liver inflammation than those with a normal eGFR, whilst there was no significant difference in mean liver fibrosis score between these two groups. We also found that in both Asian and European countries, as the liver fibrosis score gradually increased, the eGFR level gradually decreased, which was consistent with our previous study [15]. Furthermore, our regression analyses also showed that eGFR level was inversely associated with liver fibrosis severity only in the Asian MASLD population (but not in the European countries), after adjusting for common renal risk factors. This is consistent with a previous study, which revealed a geographical difference in the correlation between MASLD and risk of CKD that might be weakened in a high socio‐demographic index region [36]. A possible explanation is that Europeans generally have greater muscle mass than Asian individuals [37, 38, 39], which may result in higher serum creatinine concentrations and consequently lower eGFR levels. Additionally, the older age of our European participants when compared with Asian participants could also contribute to this finding. Our analyses found that China, Japan, and France showed consistent results, supporting a significant inverse association between eGFR and the severity of liver fibrosis. In contrast, we did not observe any association in Malaysia, Germany, Romania, and the UK. This latter finding is not consistent with previous reports [15, 19]. There are no relevant studies to support or explain our conclusion, but we suspect that this might be partly due to an insufficient sample size in some countries. We will repeat the analysis when we expand the population in future follow‐up studies.

The major strength of our multinational study is that this is the first large study to systematically describe the geographical variations of eGFR in patients with biopsy‐proven MASLD in different countries and regions worldwide. Thus, the data provide a substantial contribution to global research on eGFR in MASLD in different ethnic groups. However, the current study also has some important limitations. First, the retrospective cross‐sectional design of the study does not allow us to draw any conclusion about causality of the observed associations. A long‐term follow‐up of these participants is ongoing in a larger subsequent study to validate our findings. Second, there may have been a selection bias since we enrolled only participants with MASLD who had metabolic risk factors, but other factors, such as alcoholic liver disease, were excluded. In addition, there is a potential sample mismatch and lack of uniform inclusion and exclusion criteria across different centers and countries, which might also have introduced a bias. Third, this study did not have detailed information about albumin excretion rate, serum cystatin C concentrations, medication use or lifestyle variables. Therefore, we only calculated eGFR by using the serum creatinine‐based CKD‐EPI equation and could not accurately refine the stages of CKD for further analysis. Fourth, due to geographical and resource constraints, it was not feasible to implement central liver biopsy readings, which may have introduced some degree of reading variability or bias. We are planning to achieve a unified pathology center for the review of liver biopsy samples in future studies, thereby strengthening the validity of research outcomes. Finally, although we included data from seven countries, the sample sizes in some of these countries could be too small to accurately reflect the actual data in these groups (particularly in European countries). This disparity might lead to altered conclusions and we will continue to increase the sample size for a subsequent study.

In conclusion, our multinational study is the first to describe the geographical variations of eGFR levels in a large cohort of adults with biopsy‐confirmed MASLD. In this large multinational study, we found that the European population with MASLD had lower eGFR levels and more severe liver fibrosis than the Asian population. China had the highest mean eGFR levels, while Romania had the lowest eGFR levels. eGFR levels were closely associated with the histological severity of liver fibrosis, especially in the Asian population with MASLD. Our findings provide the basis for further investigation of the burden of MASLD on CKD risk in different countries.

## 4 Materials and Methods

### 4.1 Study Design

We performed a retrospective multinational cohort study by collecting data from 3775 participants from 34 centers in seven countries who underwent liver biopsy examination due to persistently elevated serum liver enzyme levels or imaging findings of hepatic steatosis. To better elucidate the role of metabolism in the association between MASLD and eGFR, we included only participants with biopsy‐proven MASLD and who did not have any acute kidney injury. As shown in Figure 1, 467 subjects were excluded from the analysis due to the following reasons: (1) alcohol‐related liver disease (*n* = 394), (2) age < 18 years old (*n* = 55), (3) missing data on liver fibrosis score (*n* = 3), (4) missing serum creatinine data (*n* = 8), (5) unfeasible creatinine results (*n* = 4), and (6) missing data on metabolic risk factors for the diagnosis of MASLD (*n* = 3). As a result, 3308 adult participants with biopsy‐confirmed MASLD were included in the final analysis.

### 4.2 Data Collection

In all participants, we collected clinical data on age, sex, height, body weight, waist circumference, hypertension, diabetes, platelet counts, blood glucose, creatinine, aspartate aminotransferase (AST) and ALT. BMI was calculated by dividing weight by height squared. Obesity was defined as BMI ≥ 30 kg/m^2^ in Europeans, BMI ≥ 28 kg/m^2^ in Asians [40]. eGFR was calculated using the CKD‐epidemiology collaboration (CKD‐EPI) equation, which is as follows: eGFR = 141 × min (Scr/*κ*,1)^α^ × max (Scr/*κ*,1)^−1.209^ × 0.993^Age^ × 1.018 [if female], where Scr is serum creatinine, *κ* is 0.7 for females and 0.9 for males, *α* is −0.329 for females and −0.411 for males, min indicates the minimum of Scr/*κ* or 1, and max indicates the maximum of Scr/*κ* or 1 [41, 42]. Next, we stratified our participants into two groups according to their eGFR levels, i.e., subjects with normal eGFR (≥90 mL/min/1.73 m^2^) and those with declined eGFR (<90 mL/min/1.73 m^2^).

### 4.3 Liver Biopsy Examination

The NASH Clinical Research Network (NASH CRN) scoring system and the Brunt system were used to assess the histopathological lesions [43, 44]. MASLD was defined by evidence of hepatic steatosis on liver biopsy affecting at least 5% of hepatocytes in combination with at least one of overweight or obesity, T2DM, or evidence of metabolic dysregulation for women who consume <140 g/week of alcohol and for men who consume <210 g/week and have no other known causes of hepatic steatosis. The presence of metabolic dysregulation among lean/normal weight individuals with hepatic steatosis who did not have T2DM was defined by previous study [15]. Significant steatosis, inflammation, ballooning, and fibrosis were histologically defined using a uniform standard with a pathological score ≥2 [15].

### 4.4 Statistical Analysis

Continuous variables were expressed as means ± standard deviation (SD), and categorical variables were expressed as counts or percentages (%). The one‐way analysis of variance was used for continuous variables, and the chi‐square test was used for categorical variables. Linear and binary logistic regression analyses were used to test the association between eGFR level and liver fibrosis scores on histology after adjusting for confounding variables, such as sex, BMI, waist circumference, serum transaminases, diabetes, and hypertension. The regression coefficients beta (*β*) and ORs were calculated with 95% CI. When a variable with missing values was included, cases with missing data on that variable were automatically excluded from the analysis. All tests were two‐sided, a *p* value ≤ 0.05 was considered statistically significant. All statistical analyses were performed using SPSS 22.0 (SPSS, Chicago, IL).

## Author Contributions

MHZ, DQS, and JZ were involved in study design and data interpretation and verification. JZ performed data analysis and wrote the manuscript. Data collection was done by FEM, XYX, DJ, HL, XC, JC, TO, TS, YZ, CL, BZ, MY, AN, JS, JZ, SM, WKC, FH, CYY, SL, AD, VdL, MLP, JG, ZL, HT, JJ, PJE, LX, YL, YN, HD, JN, XY, QY, QLZ, YZ, JFLC, CL, JL, LL, JW, FJ, JC, YX, GT, CDB, YF, JHZ, GO, MP, DQS, MHZ. GT, CDB, and MP conducted critical revision and writing of the manuscript. All authors reviewed and commented on the manuscript and approved the final version.

## Funding

This work was supported by grants from the National Natural Science Foundation of China (82570853, 82500873, 82370577, 82070588) and supported by China Postdoctoral Science Foundation (2023M732681). Dan‐Qin Sun is supported in part by grants from Top Talent Support Program for young and middle‐aged people of Wuxi Health Committee (BJ2023023) and scientific technological innovation and venture capital fund in Wuxi (Y20232011). GT is supported in part by grants from the School of Medicine, University of Verona, Verona, Italy. CDB is supported in part by the Southampton NIHR Biomedical Research Centre (NIHR203319), UK.

## Ethics Statement

This study adhered to the principles of the Helsinki Declaration and the need for informed written consent was waived due to the retrospective nature of the study. This study was approved by the guidelines of the ethics committee of the the First Affiliated Hospital of Wenzhou Medical University (No. 2016‐246).

## Conflicts of Interest

WKC has served as a consultant or advisory board member for Roche, Abbvie, Boehringer Ingelheim, and Novo Nordisk; and a speaker for Echosens, Viatris, and Hisky Medical. Victor de Lédinghen has served as a consultant or advisory committee member for Gilead Sciences, Intercept, Alfasigma, Orphalan, and Mindray; and a speaker for AbbVie, Echosens, Gilead Sciences, Hologic, Tillotts, Orphalan, and Janssen. Fanpu Ji reports lecture fees from Gilead Sciences, MSD, and Ascletis and he is a consultant for Gilead and MSD. Others have no conflict of interest. CDB has received grant support from Echosens.

## Data Availability

The datasets generated and analyzed during the current study are not publicly available because of other unpublished data but are available from the corresponding author on reasonable request.

## References

[1] G. Feng , L. Valenti , V. W. Wong , et al., “Recompensation in Cirrhosis: Unravelling the Evolving Natural History of Nonalcoholic Fatty Liver Disease,” Nature Reviews Gastroenterology & Hepatology 21, no. 1 (2024): 46–56. 37798441 10.1038/s41575-023-00846-4

[2] G. E. H. Lim , A. Tang , C. H. Ng , et al., “An Observational Data Meta‐analysis on the Differences in Prevalence and Risk Factors between MAFLD vs NAFLD,” Clinical Gastroenterology and Hepatology: the Official Clinical Practice Journal of the American Gastroenterological Association 21, no. 3 (2023): 619–629. e617. 34871813 10.1016/j.cgh.2021.11.038

[3] L. Miao , G. Targher , C. D. Byrne , Y. Y. Cao , and M. H. Zheng , “Current Status and Future Trends of the Global Burden of MASLD,” Trends in Endocrinology and Metabolism: TEM 35, no. 8 (2024): 697–707. 38429161 10.1016/j.tem.2024.02.007

[4] M. Eslam , A. J. Sanyal , and J. George , “MAFLD: A Consensus‐Driven Proposed Nomenclature for Metabolic Associated Fatty Liver Disease,” Gastroenterology 158, no. 7 (2020): 1999–2014. e1991. 32044314 10.1053/j.gastro.2019.11.312

[5] A. De , N. Bhagat , M. Mehta , S. Taneja , and A. Duseja , “Metabolic Dysfunction‐associated Steatotic Liver Disease (MASLD) Definition Is Better Than MAFLD Criteria for Lean Patients With NAFLD,” Journal of Hepatology 80, no. 2 (2024): e61–e62. 37558135 10.1016/j.jhep.2023.07.031

[6] K. I. Zheng , D. Q. Sun , Y. Jin , P. W. Zhu , and M. H. Zheng , “Clinical Utility of the MAFLD Definition,” Journal of Hepatology 74, no. 4 (2021): 989–991. 33347953 10.1016/j.jhep.2020.12.016

[7] J. Zhou , D.‐Q. Sun , G. Targher , et al., “Metabolic Dysfunction‐associated Fatty Liver Disease Increases Risk of Chronic Kidney Disease: A Systematic Review and Meta‐analysis,” Egastroenterology 1, no. 1 (2023): e100005. 39944252 10.1136/egastro-2023-100005 PMC11770460

[8] V. H. Nguyen , M. H. Le , R. C. Cheung , and M. H. Nguyen , “Differential Clinical Characteristics and Mortality Outcomes in Persons with NAFLD and/or MAFLD,” Clinical Gastroenterology and Hepatology: the Official Clinical Practice Journal of the American Gastroenterological Association 19, no. 10 (2021): 2172–2181. e2176. 34033923 10.1016/j.cgh.2021.05.029

[9] T. Y. Wang , R. F. Wang , Z. Y. Bu , et al., “Association of Metabolic Dysfunction‐associated Fatty Liver Disease With Kidney Disease,” Nature Reviews Nephrology 18, no. 4 (2022): 259–268. 35013596 10.1038/s41581-021-00519-y

[10] J. Zhao , L. Liu , Y. Y. Cao , et al., “MAFLD as Part of Systemic Metabolic Dysregulation,” Hepatology International 18, Suppl no. 2 (2024): 834–847. 38594474 10.1007/s12072-024-10660-y

[11] J. Bilson , T. J. Hydes , D. McDonnell , et al., “Impact of Metabolic Syndrome Traits on Kidney Disease Risk in Individuals With MASLD: A UK Biobank Study,” Liver International: Official Journal of the International Association for the Study of the Liver 45, no. 4 (2025): e16159. 39548715 10.1111/liv.16159 PMC11897864

[12] D. J. Cuthbertson , O. J. Kennedy , J. Bilson , et al., “Impact of Metabolic Dysfunction Severity in Steatotic Liver Disease and Its Interaction With Liver Fibrosis on all‐cause Mortality and Multiple Hepatic and Extra‐hepatic Outcomes,” Metabolism: Clinical and Experimental 170 (2025): 156306. 40414560 10.1016/j.metabol.2025.156306

[13] G. Collaboration , “Global, Regional, and National Burden of Chronic Kidney Disease, 1990‐2017: A Systematic Analysis for the Global Burden of Disease Study 2017,” Lancet (London, England) 395, no. 10225 (2020): 709–733. 32061315 10.1016/S0140-6736(20)30045-3 PMC7049905

[14] J. Hu , R. Ke , W. Teixeira , et al., “Global, Regional, and National Burden of CKD due to Glomerulonephritis From 1990 to 2019: A Systematic Analysis From the Global Burden of Disease Study 2019,” Clinical Journal of the American Society of Nephrology: CJASN 18, no. 1 (2023): 60–71. 36719159 10.2215/CJN.0000000000000017 PMC10101559

[15] D. Q. Sun , Y. Jin , and T. Y. Wang , “MAFLD and Risk of CKD,” Metabolism: Clinical and Experimental 115 (2021): 154433. 33212070 10.1016/j.metabol.2020.154433

[16] Y. Liang , H. Chen , Y. Liu , et al., “Association of MAFLD with Diabetes, Chronic Kidney Disease, and Cardiovascular Disease: A 4.6‐Year Cohort Study in China,” The Journal of Clinical Endocrinology and Metabolism 107, no. 1 (2022): 88–97. 34508601 10.1210/clinem/dgab641 PMC8684479

[17] S. Chen , J. Pang , R. Huang , H. Xue , and X. Chen , “Association of MAFLD With End‐stage Kidney Disease: A Prospective Study of 337,783 UK Biobank Participants,” Hepatology International 17, no. 3 (2023): 595–605. 36809487 10.1007/s12072-023-10486-0

[18] C. Sun , G. B. Goh , W. C. Chow , et al., “Prevalence and Risk Factors for Impaired Renal Function Among Asian Patients With Nonalcoholic Fatty Liver Disease,” Hepatobiliary & Pancreatic Diseases International: HBPD INT 23, no. 3 (2023): 241–248. 37620227 10.1016/j.hbpd.2023.08.004

[19] D. Q. Sun , G. Targher , C. D. Byrne , et al., “An International Delphi Consensus Statement on Metabolic Dysfunction‐associated Fatty Liver Disease and Risk of Chronic Kidney Disease,” Hepatobiliary Surgery and Nutrition 12, no. 3 (2023): 386–403. 37351121 10.21037/hbsn-22-421 PMC10282675

[20] L. A. Inker and S. Titan , “Measurement and Estimation of GFR for Use in Clinical Practice: Core Curriculum 2021,” American Journal of Kidney Diseases: the Official Journal of the National Kidney Foundation 78, no. 5 (2021): 736–749. 34518032 10.1053/j.ajkd.2021.04.016

[21] J. Coresh , T. C. Turin , K. Matsushita , et al., “Decline in Estimated Glomerular Filtration Rate and Subsequent Risk of End‐stage Renal Disease and Mortality,” Jama 311, no. 24 (2014): 2518–2531. 24892770 10.1001/jama.2014.6634 PMC4172342

[22] A. Ramezankhani , F. Azizi , and F. Hadaegh , “Association Between Estimated Glomerular Filtration Rate Slope and Cardiovascular Disease Among Individuals With and Without Diabetes: A Prospective Cohort Study,” Cardiovascular Diabetology 22, no. 1 (2023): 270. 37794456 10.1186/s12933-023-02008-x PMC10552420

[23] L. Liu , M. Zhu , Q. Meng , et al., “Association Between Kidney Function and the Risk of Cancer: Results From the China Health and Retirement Longitudinal Study (CHARLS),” Journal of Cancer 11, no. 21 (2020): 6429–6436. 33033526 10.7150/jca.47175 PMC7532505

[24] D. Q. Sun , J. Q. Shen , X. F. Tong , et al., “Liver Fibrosis Progression Analyzed With AI Predicts Renal Decline,” JHEP Reports: Innovation in Hepatology 7, no. 5 (2025): 101358. 40321195 10.1016/j.jhepr.2025.101358 PMC12048807

[25] K. E. Chan , T. J. L. Koh , A. S. P. Tang , et al., “Global Prevalence and Clinical Characteristics of Metabolic‐associated Fatty Liver Disease: A Meta‐Analysis and Systematic Review of 10 739 607 Individuals,” The Journal of Clinical Endocrinology and Metabolism 107, no. 9 (2022): 2691–2700. 35587339 10.1210/clinem/dgac321

[26] J. Liu , I. Ayada , X. Zhang , et al., “Estimating Global Prevalence of Metabolic Dysfunction‐Associated Fatty Liver Disease in Overweight or Obese Adults,” Clinical Gastroenterology and Hepatology: the Official Clinical Practice Journal of the American Gastroenterological Association 20, no. 3 (2022): e573–e582. 33618024 10.1016/j.cgh.2021.02.030

[27] W. Zhang , W. J. Song , W. Chen , et al., “Metabolic Dysfunction‐associated Steatotic Liver Disease‐related Hepatic Fibrosis Increases Risk of Insulin Resistance, Type 2 Diabetes, and Chronic Kidney Disease,” European Journal of Gastroenterology & Hepatology 36, no. 6 (2024): 802–810. 38526946 10.1097/MEG.0000000000002767 PMC11045407

[28] K. Brück , V. S. Stel , G. Gambaro , et al., “CKD Prevalence Varies Across the European General Population,” Journal of the American Society of Nephrology: JASN 27, no. 7 (2016): 2135–2147. 26701975 10.1681/ASN.2015050542 PMC4926978

[29] P. A. Kavsak and K. Nouri , “Low‐risk Cutoff of 90 Ml/Min/1.73 M(2) for the Estimated Glomerular Filtration Rate and the Importance of the Equation for Patients With Acute Coronary Syndrome,” Clinica Chimica Acta; International Journal of Clinical Chemistry 523 (2021): 532–533. 34767793 10.1016/j.cca.2021.11.010

[30] M. L. Caramori , P. Fioretto , and M. Mauer , “Low Glomerular Filtration Rate in Normoalbuminuric Type 1 Diabetic Patients: An Indicator of More Advanced Glomerular Lesions,” Diabetes 52, no. 4 (2003): 1036–1040. 12663477 10.2337/diabetes.52.4.1036

[31] Y. Hashimoto , M. Tanaka , H. Okada , et al., “Metabolically Healthy Obesity and Risk of Incident CKD,” Clinical Journal of the American Society of Nephrology: CJASN 10, no. 4 (2015): 578–583. 25635035 10.2215/CJN.08980914 PMC4386260

[32] K. Chintam and A. R. Chang , “Strategies to Treat Obesity in Patients with CKD,” American Journal of Kidney Diseases: the Official Journal of the National Kidney Foundation 77, no. 3 (2021): 427–439. 33075388 10.1053/j.ajkd.2020.08.016 PMC7904606

[33] I. Helal , G. M. Fick‐Brosnahan , B. Reed‐Gitomer , and R. W. Schrier , “Glomerular Hyperfiltration: Definitions, Mechanisms and Clinical Implications,” Nature Reviews Nephrology 8, no. 5 (2012): 293–300. 22349487 10.1038/nrneph.2012.19

[34] D. C. Rockey , S. H. Caldwell , Z. D. Goodman , R. C. Nelson , and A. D. Smith , “Liver Biopsy,” Hepatology (Baltimore, Md) 49, no. 3 (2009): 1017–1044. 10.1002/hep.22742 19243014

[35] D. Q. Sun , F. Z. Ye , H. T. Kani , et al., “Higher Liver Stiffness Scores Are Associated With Early Kidney Dysfunction in Patients With Histologically Proven Non‐cirrhotic NAFLD,” Diabetes & Metabolism 46, no. 4 (2020): 288–295. 31786360 10.1016/j.diabet.2019.11.003

[36] J. Bai , L. Zhang , M. Zhang , Y. Hao , Z. Yi , and Y. Zhou , “Regional Insights Into the Relationship Between Metabolic Associated Steatotic Liver Disease and Chronic Kidney Disease: A Socioeconomic Perspective on Disease Correlation,” BMC Public Health [Electronic Resource] 25, no. 1 (2025): 993. 40082841 10.1186/s12889-025-22188-3 PMC11907813

[37] S. Chen , X. Xu , H. Gong , et al., “Global Epidemiological Features and Impact of Osteosarcopenia: A Comprehensive Meta‐analysis and Systematic Review,” Journal of Cachexia, Sarcopenia and Muscle 15, no. 1 (2024): 8–20. 38086772 10.1002/jcsm.13392 PMC10834350

[38] T. Abe , M. G. Bemben , M. Kondo , Y. Kawakami , and T. Fukunaga , “Comparison of Skeletal Muscle Mass to Fat‐free Mass Ratios Among Different Ethnic Groups,” The Journal of Nutrition, Health & Aging 16, no. 6 (2012): 534–538.10.1007/s12603-012-0015-222659992

[39] I. Janssen , S. B. Heymsfield , R. N. Baumgartner , and R. Ross , “Estimation of Skeletal Muscle Mass by Bioelectrical Impedance Analysis,” Journal of Applied Physiology (Bethesda, Md: 1985) 89, no. 2 (2000): 465–471. 10926627 10.1152/jappl.2000.89.2.465

[40] Z. M. Younossi , S. Zelber‐Sagi , J. V. Lazarus , et al., “Global Consensus Recommendations for Metabolic Dysfunction‐Associated Steatotic Liver Disease and Steatohepatitis,” Gastroenterology 169, no. 5 (2025): 1017–1032. e1012. 40222485 10.1053/j.gastro.2025.02.044

[41] A. S. Levey , L. A. Stevens , C. H. Schmid , et al., “A New Equation to Estimate Glomerular Filtration Rate,” Annals of Internal Medicine 150, no. 9 (2009): 604–612. 19414839 10.7326/0003-4819-150-9-200905050-00006 PMC2763564

[42] C. Delgado , M. Baweja , D. C. Crews , et al., “A Unifying Approach for GFR Estimation: Recommendations of the NKF‐ASN Task Force on Reassessing the Inclusion of Race in Diagnosing Kidney Disease,” American Journal of Kidney Diseases: the Official Journal of the National Kidney Foundation 79, no. 2 (2022): 268–288. e261. 34563581 10.1053/j.ajkd.2021.08.003

[43] A. J. Sanyal , R. Loomba , Q. M. Anstee , et al., “Utility of Pathologist Panels for Achieving Consensus in NASH Histologic Scoring in Clinical Trials: Data From a Phase 3 Study,” Hepatology Communications 8, no. 1 (2024): e0325. 38126958 10.1097/HC9.0000000000000325 PMC10749704

[44] D. E. Kleiner , E. M. Brunt , M. Van Natta , et al., “Design and Validation of a Histological Scoring System for Nonalcoholic Fatty Liver Disease,” Hepatology (Baltimore, Md) 41, no. 6 (2005): 1313–1321. 10.1002/hep.20701 15915461

